# Supplementary figures and images for: The Role of Interstitial Fluid Pressure in Cerebral Porous Biomaterial Integration
Source: Brain Sci. 2022 Mar 22;12(4):417. doi: 10.3390/brainsci12040417 (PMC9040716; doi:10.3390/brainsci12040417)

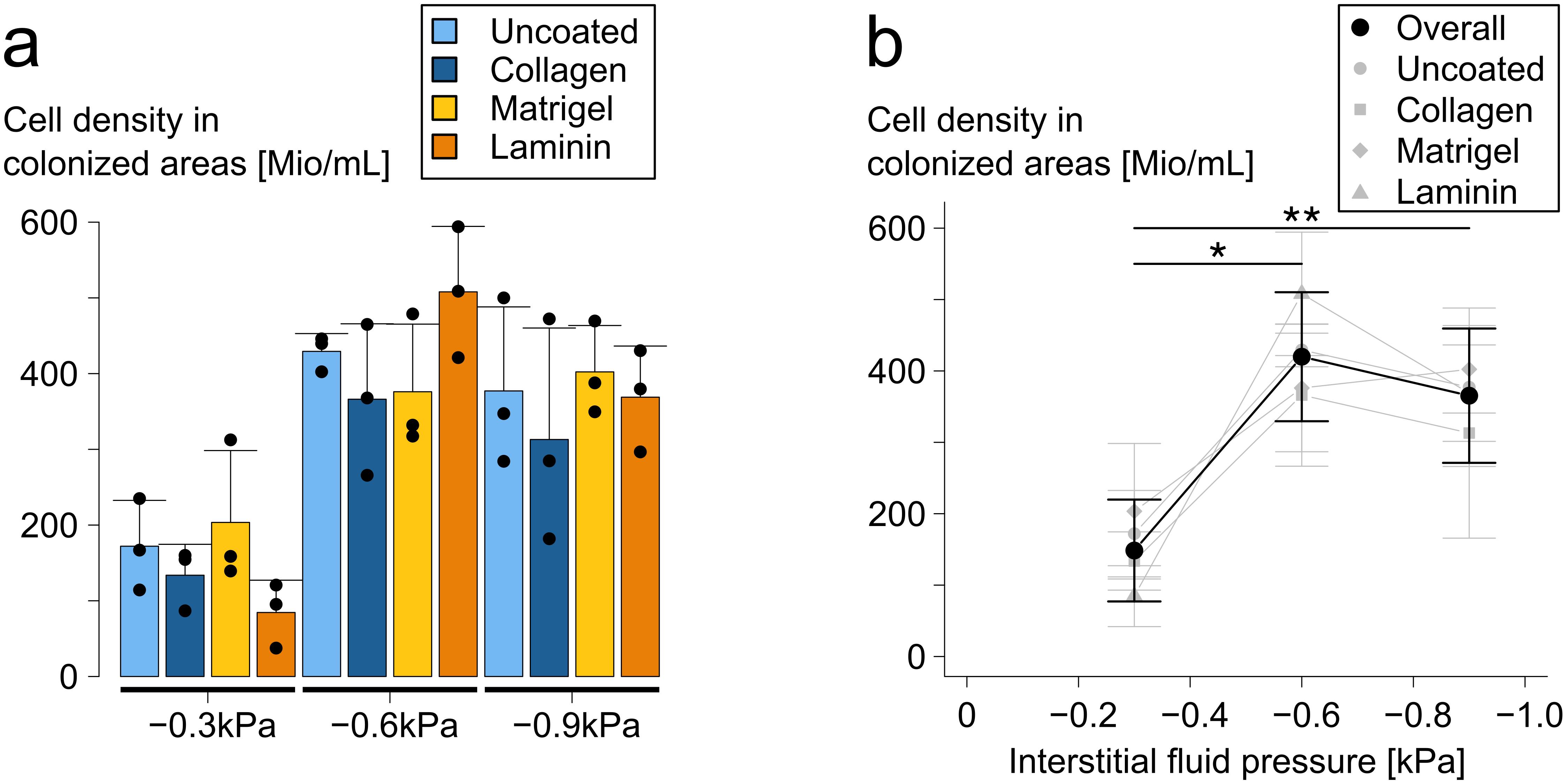

Supplement: Supplementary file 1 [file brainsci-12-00417-s001.zip › Supplementary Figure S1.jpg]

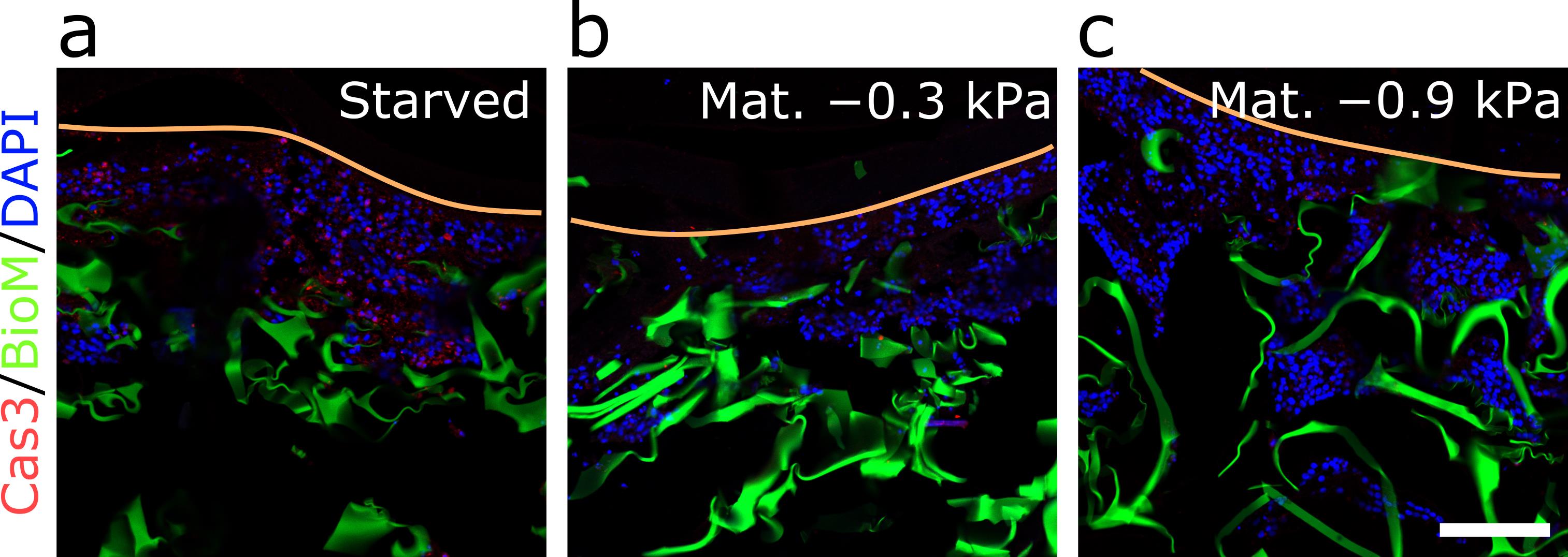

Supplement: Supplementary file 1 [file brainsci-12-00417-s001.zip › Supplementary Figure S2.jpg]
